# Heme oxygenase-1 in pregnancy and cancer: similarities in cellular invasion, cytoprotection, angiogenesis, and immunomodulation

**DOI:** 10.3389/fphar.2014.00295

**Published:** 2015-01-14

**Authors:** Hui Zhao, Maide Ozen, Ronald J. Wong, David K. Stevenson

**Affiliations:** Department of Pediatrics, Division of Neonatal and Developmental Medicine, Stanford University School of MedicineStanford, CA, USA

**Keywords:** Placenta, trophoblast invasion, angiogenesis, immunosuppression, tolerogenic dendritic cells (tDC), alternatively activated macrophage (M2)

## Abstract

Pregnancy can be defined as a “permissible” process, where a semi-allogeneic fetus and placenta are allowed to grow and survive within the mother. Similarly, in tumor growth, antigen-specific malignant cells proliferate and evade into normal tissues of the host. The microenvironments of the placenta and tumors are amazingly comparable, sharing similar mechanisms exploited by fetal or cancer cells with regard to surviving in a hypoxic microenvironment, invading tissues via degradation and vasculogenesis, and escaping host attack through immune privilege. Heme oxygease-1 (HO-1) is a stress-response protein that has antioxidative, anti-apoptotic, pro-angiogenic, and anti-inflammatory properties. Although a large volume of research has been published in recent years investigating the possible role(s) of HO-1 in pregnancy and in cancer development, the molecular mechanisms that regulate these “yin-yang” processes have still not been fully elucidated. Here, we summarize and compare pregnancy and cancer development, focusing primarily on the function of HO-1 in cellular invasion, cytoprotection, angiogenesis, and immunomodulation. Due to the similarities of both processes, a thorough understanding of the molecular mechanisms of each process may reveal and guide the development of new approaches to prevent not only pregnancy disorders; but also, to study cancer.

## INTRODUCTION

**FIGURE 1:**
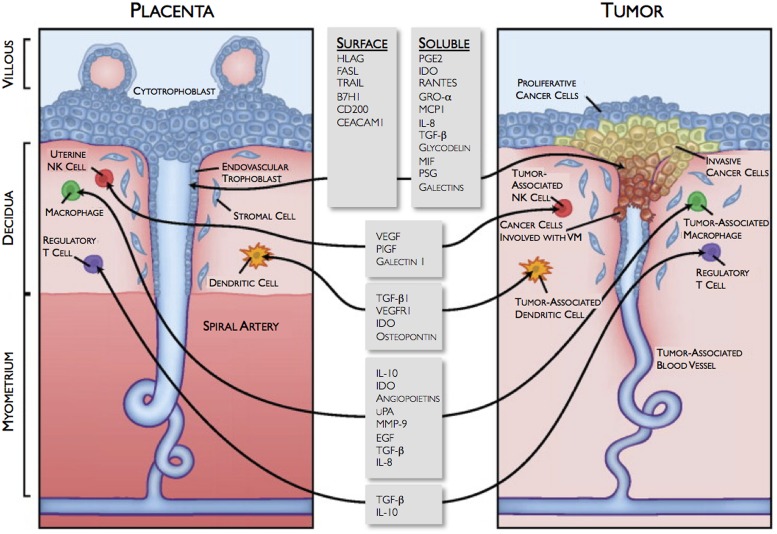
**Similarities between the fetomaternal interface and tumor microenvironment.** VM, vasculogenic mimicry. Reprinted with permission from Holtan et al. (2009) ©2009 Mayo Foundation for Medical Education and Research.

The development of the placenta is a highly regulated and complex *physiologic* process that is required to ensure proper fetal development, and the creation of life. In contrast, the formation of cancer is a *pathologic* process, characterized by uncontrolled growth of malignant cells that may ultimately lead to death. Even with these obvious differences, in some aspects, these two “yin-yang” processes actually are very similar, incorporating comparable mechanisms regulating cell growth, cell invasion, neovascularization, and immunotolerance (Holtan et al., 2009; **Figure 1**). In early placental development, trophoblast cells, originating from the developing embryo, implant in the uterine wall and encounter extremely harsh conditions, such as extreme hypoxia (∼1–2% oxygen), lack of a vasculature supply, and potential immune attacks from the maternal immune system. Interestingly, this environment is similar to that for an invading tumor. Malignant cells must also aggressively invade into normal tissue, establish a vasculature system, and defend against a host’s immune response. Somehow, both trophoblast, and malignant cells are able to successfully survive and grow (Holtan et al., 2009).

Heme oxygenase (HO) is a gene that is conserved across all biological kingdoms (animals, plants, and bacteria), and its role in heme degradation has been well understood. There are 3 known isoforms: the inducible HO-1, the constitutively expressed HO-2, and the pseudogene HO-3 (Cruse and Maines, 1988; Maines, 1988, 1997; McCoubrey et al., 1997). HO-1 can be upregulated by its substrate heme and also by heat shock, heavy metals, endotoxin, prostaglandins, inflammatory cytokines, etc. Therefore, it is referred to as a stress-response protein. HO-1 is ubiquitously expressed, however, its expression level and the associated functions vary from cell type to cell type. HO and its metabolites, carbon monoxide (CO), iron, and bilirubin, also exhibit significant antioxidative, cytoprotective, pro-angiogenic, neurotransmitting, and anti-inflammatory properties (Abraham et al., 1988; Marks et al., 1991; Choi and Alam, 1996). Its beneficial effects have been observed in atherosclerosis, diabetes, ischemia/reperfusion injury, as well as in organ transplantation (Shibahara et al., 2003; Exner et al., 2004).

The HO/CO system is believed to be important in the maintenance of a healthy pregnancy. Several investigators have reported an association between a low expression of HO-1 with pregnancy disorders. For example, immunohistochemical staining of HO-1 in human placentas was lower in pre-eclamptic patients compared to pregnant controls (Ahmed et al., 2000). End-tidal breath CO levels were lower in hypertensive pregnant women, especially those with severe pre-eclampsia, than pregnant healthy women (Baum et al., 2000; Kreiser et al., 2004). Reduced HO-1 expression was also found in mouse models of spontaneous abortions (Zenclussen et al., 2002, 2006b).

Similarly, HO-1 has been linked with tumor induction, growth, and metastasis. Induction of HO-1 expression has been observed in tumors, such as lymphosarcomas, prostatic cancers, glioblastomas, hepatomas, melanomas, pancreatic cancers, and chronic myeloid leukemias (Was et al., 2010). Tumors exhibit strong neovascularization and massive hemorrhaging, and therefore can release significant amounts of heme, which can then directly induce HO-1 expression and confer cytoprotection from oxidative injury. HO-1 is also involved in tumor angiogenesis and stimulating tumor-associated macrophages (TAMs; Was et al., 2006). Therefore, HO-1 may be involved in tumor survival and progression.

This review summarizes the common properties shared by the processes of placental and cancer development, emphasizing the role of HO-1 on cytoprotection, angiogenesis, and immune privilege. Mechanisms used by malignant cells to proliferate, invade, and immunosuppress may also apply to those used by trophoblast cells in the development of the fetomaternal interface. The understanding of these two clinically different, yet biologically similar, processes may lead us to develop new approaches to prevent pregnancy disorders related to placental disorders.

## HO-1 EXPRESSION AND INVASIVENESS OF TROPHOBLAST AND TUMOR CELLS

HO-1 expression has been studied in both human and mouse placentas. In human placental tissue, the results have been inconsistent (Lyall et al., 2000; McLean et al., 2000; Yoshiki et al., 2000; McLaughlin et al., 2003; Bilban et al., 2009), very likely due to the intra- and inter-tissue heterogeneity and different gestational ages studied. Due to the difficulties of human sample collection, most studies were performed using either term or preterm-delivered placentas. In addition, for most cases, the tissue collected was only from the placenta and without the placental bed, and used only for immunohistochemical studies.

To date, the most systematic study is from Lyall et al. (2000), who collected both the placenta and placental bed tissues from biopsies done in first two trimesters (8 and 19 weeks) as well as at term using a transcervical sampling technique. They found that in villous tissue (placenta), HO-1 was low and was not gestational age-dependent, while HO-2 displayed a spatial and temporal pattern of expression: HO-2 was predominant in syncytiotrophoblast in the first trimester and decreased at term, while endothelial immunostaining was weak in the first trimester, but increased by term. However, within the placental bed, both HO-1 and HO-2 were intensely expressed in extravillous trophoblasts (EVTs, with high invasiveness), but absent from the proximal cytotrophoblast (CTBs, with less invasiveness) layers of cell column (Lyall et al., 2000). EVTs are highly proliferative and invasive in nature. They are involved in the attachment of the placenta to the decidua (uterine wall) by migrating through the syncytiotrophoblast and also in the modification of the spiral arteries. The authors believe that since HO-1 and HO-2 is highly expressed in EVTs, HO plays a role in trophoblast invasion and transformation of spiral arteries.

However, Bilban et al. (2009) also collected first trimester human placental tissues and compared the mRNA expression profiles of EVTs to the less invasive CTBs. They found that HO-1 was actually downregulated in EVTs. In addition, a high expression of HO-1 in the proliferative, Ki67-positive cell column was detected, in contrast to the low levels observed in non-cycling, Ki67-negative, invasive EVTs. They further showed that HO-1 can negatively regulate the motility of trophoblasts by acting via the nuclear hormone peroxisome proliferator-activated receptor (PPAR)-γ. The contradictory results from the two groups may be due to the time of placental bed sample collection. During the first 10–12 weeks of gestation, EVTs form “plugs” that can prevent maternal blood flow into the intervillous space and creating a hypoxic environment. During their migration away from the villi, EVTs differentiate into an invasive phenotype. Dynamic changes of oxygen level, availability of heme, and variations in tissue sampling may affect downstream results and account for the conflicting findings. Despite this, HO-1 is still believed to be critically involved in mediating the differentiation, invasion, and motility of EVTs during the first trimester of pregnancy.

Unlike that of the human, invasive trophoblasts in the mouse placenta have very shallow invasion into the decidua. Therefore, it is understandable that there is a weaker staining of HO-1 in the mouse decidua, which is mostly confined to the spongiotrophoblast layer in the junction zone, which serves as the interface between the maternal and embryonic sides of the placenta (Watanabe et al., 2004; Zhao et al., 2009). It is not clear if invasive trophoblasts or functional equivalent cells of human EVTs are located in the junction zone in the mouse placenta. Using histological examination as well as 3-D images from casted placentas, Zhao et al. (2009, 2011), Wong et al. (2012) observed that in HO-1 heterozygote (Het, HO-1^+/-^) placentas, there is an increase in apoptosis that leads to markedly thinner junction zones compared to those in wild-type (Wt) placentas. Therefore, in the mouse placenta, cells in the junction zone may be interesting candidates for investigating the function of HO-1 in decidual formation and spiral artery remodeling.

Interestingly, invasive trophoblast cells are strikingly similar to cancer cells in their capacities to proliferate, migrate, and die, making comparisons to cancer development very compelling. Both trophoblasts and cancer cells are rapidly dividing cells, where HO-1 is abundantly expressed. Many human tumors produce HO-1 and its expression is usually higher in cancer cells than in surrounding healthy tissues (Was et al., 2010). HO-1 is localized in either tumor cells or macrophages or both, but its exact location in transformed tissues depends on the type of tumor as well as its stage of development. For example, in human melanomas (Torisu-Itakura et al., 2000) or gliomas (Deininger et al., 2000), HO-1 was almost exclusively expressed in macrophages, which accumulated around the necrotic area. In human pancreatic carcinomas, HO-1 immunoreactivity was found in both cancer and immune cells (Berberat et al., 2005). In rat hepatomas, HO-1 was found only in tumor cells (Doi et al., 1999).

In addition, a high expression of HO-1 may be associated with poor prognosis in patients with non-small cell lung cancers (Tsai et al., 2012). In contrast, patients with a high expression of HO-1 and colorectal cancers have a favorable prognosis, and those with oral squamous cell carcinomas have a low risk of lymph node metastases (Noh et al., 2013). The association of HO-1 and tumor cell invasion is also inconsistent and appears to vary among tumor types. For example, HO-1 inhibits invasion of breast cancer by suppressing the expression of matrix metalloproteinase-9 (MMP-9; Lin et al., 2008), but promotes gastric cancer invasion via the sonic hedgehop signaling pathway (Xu et al., 2012). Therefore, the role of HO-1 in human malignant tumor growth may vary depending upon tumor type.

## EFFECTS OF HO-1 ON CYTOPROTECTION IN THE HYPOXIC ENVIRONMENT

HO-1 is a well-known strong antioxidant that has been shown to protect various cell types from oxidative damage and to reduce the rate of apoptosis. The most convincing data describing these cytoprotective properties are from studies from Poss and Tonegawa (1997), who first established the HO-1 knockout (HO-1^-/-^) mouse. Using this mouse model, they showed that HO-1 deficiency leads to severe oxidative stress with elevated lipid peroxidation, cardiovascular damage, and progressive chronic inflammation in the kidney and liver. Moreover, fibroblasts isolated from HO-1^-/-^ mice showed increased production of reactive oxygen species (ROS) and reduced cell viability when exposed to various oxidants (Poss and Tonegawa, 1997).

In early pregnancy, the fetal environment is extremely “hypoxic.” From 8 to 10 weeks of gestation, Oxygen (O_2_) levels in the intervillous space is around 18 mm Hg compared to 40 and 61 mm Hg in the endometrium and at the end of the third trimester, respectively. From 12 to 13 weeks of gestation, placental O_2_ levels increase to levels similar to those measured in the endometrium (Rodesch et al., 1992; Jaffe et al., 1997; Burton et al., 1999). In first trimester, an hypoxic environment may be important in the regulation of trophoblast differentiation that is mediated through a complex set of interactions between factors associated with oxidative stress, oxygen-sensing, and the release of inflammatory cytokine (James et al., 2006). It is currently unclear whether this increase of ROS production in trophoblasts is a direct result of hypoxia as these cells have the ability to control oxidative stress using several different pathways. Genes responsive to hypoxia are those that enhance O_2_ delivery, decrease O_2_ consumption, or regulate cellular metabolism.

The response of HO-1 to hypoxia or its association with the trophoblast differentiation has not been well established. On one hand, as O_2_ availability is reduced, HO activity actually decreases since molecular O_2_ is a co-factor required for heme breakdown (Appleton et al., 2002). On the other hand, although HO-1 mRNA and protein levels have been shown to increase in response to hypoxia in several organ systems, placental HO-1 expression under hypoxia is controversial. Using human term placental explants, Appleton et al. (2003) found that HO-1 levels were unaffected when the explants were exposed to different O_2_ tensions. However, George et al. (2012) have reported that 48 h of chronic hypoxic exposure down-regulated HO-1 expression in cultured rat placental villous explants. In contrast, in an established ischemia [reduced uterine perfusion pressure (RUPP]) model, they found that placental HO-1 increased compared to non-RUPP-treated controls (George et al., 2011b). Interestingly, using a rat trophoblast stem cell line, Zenclussen et al. (2011) found that HO-1 inhibition could impair cell viability and abolish their differentiation to giant cells.

During tumor proliferation, regions of the tumor may have significantly lower O_2_ concentrations than healthy tissues as they rapidly outgrows their own blood supplies. In order to support continuous growth and proliferation under these hypoxic conditions, cancer cells can alter their metabolism as well as increase their cytoprotective enzymes, such as HO-1 and its metabolite CO. Pharmacological or genetic activation of HO-1 significantly improves survival of many tumors, such as hepatomas, thyroid carcinomas, chronic myeloid leukemias, gastric carcinomas, and gliomas. In contrast, HO-1 inhibition can reduce colon carcinomas, acute myeloid leukemias, and hormone-refractory prostate cancers (Was et al., 2010). Expression of HO-1 is further elevated in response to anti-cancer treatments, such as chemotherapy, radiotherapy, and photodynamic therapy, all of which can induce hypoxia, oxidation, and pro-apoptosis or pro-necrosis in cancer cells (Was et al., 2010). Induction of HO-1 seems to counteract these treatments, hindering the effectiveness of anti-cancer therapies, and inducing resistance.

The mechanisms involved in the cytoprotective effects of HO-1 in tumor cells are still not fully known. One pathway that has been postulated is the removal of free heme. Another is the increase of cellular biliverdin/bilirubin, both strong antioxidants. In tumors, there are high free heme levels due to massive hemorrhaging and necrosis of host cells and tissues. In the fetomaternal interface, cell apoptosis is constantly occurring, but it is not clear if free heme resulting from cell death has any role in affecting HO-1 expression.

## HO-1 IN PLACENTAL VASCULATURE DEVELOPMENT

Normal placental development is a balance of angiogenesis and vasculogenesis, which is believed to be mediated by a crosstalk between different cell types. Although HO-1-deficient animals do not show any visible phenotype suggestive of vasculature defects, accumulating evidence reveals an association between HO-1 deficiency and poor angiogenesis. Direct effects of HO-1 on angiogenesis have been mostly studied in endothelial or endothelial progenitor cells (EPCs). Deramaudt et al. (1998) have shown that the overexpression of HO-1 enhances endothelial cell proliferation. Li Volti et al. (2005) showed that by inhibiting HO-1 by antisense strategies, both endothelial cell proliferation and capillary formation decrease *in vitro*, which may be associated with the cell cycle. However, the effect of HO-1 on angiogenesis can also be indirect and mediated through pro-angiogenic factors or the recruitment of EPCs. The upregulation of HO-1 (and hence CO) increases the synthesis of the pro-angiogenic factors, vascular endothelial growth factor (VEGF), monocyte chemotactic protein 1 (MCP-1 or CCL2), transforming growth factor (TGF)-β, and IL-8; and decreases production of anti-angiogenic mediators: soluble Flt-1 (sFlt-1), soluble endoglin (sEng), and CXCL10 (Cudmore et al., 2007; Deshane et al., 2007; Dulak et al., 2008; Loboda et al., 2008), to result in a stimulation of angiogenesis and vasculogenesis.

Stromal cell-derived factor-1 (SDF-1 or CXCL12) plays a major regulatory role in the migration, recruitment, and retention of EPCs to areas of ischemic injury and contributes to neovascularization. Inactivation of SDF-1 or its receptor in mice leads to intrauterine deaths due to abnormalities in vascular development (Ma et al., 1998; Ratajczak et al., 2006). The first study revealing that HO-1 is directly involved in regulating angiogenesis via the SDF-1 pathway is from the laboratory of Agarwal (Deshane et al., 2007). They demonstrated that SDF-1 upregulates HO-1 in endothelial cells through a protein kinase C-dependent, but VEGF-independent, pathway. In the absence of HO-1, SDF-1 was unable to promote endothelial tube formation and migration or induce the formation of capillary sprouts and aortic rings. Interestingly, these defects can be reversed by exogenous CO administration. In addition, they observed that the impairment of wound healing in HO-1-deficient mice is due, in part, to a reduction of EPC recruitment and capillary formation. These HO-1-deficient EPCs were unable to re-endothelialize the retina after ischemic injury (Deshane et al., 2007).

In the placenta, SDF-1 expression has been found in trophoblast cells, especially EVTs. Hanna et al. (2003) showed that SDF-1 produced by EVTs induces the specific migration of human blood CD16^-^ natural killer (NK) cells via CXCR-4 receptors. CD16^-^ NK cells are a unique subset of blood NK cells that share a similar phenotype with decidual NK (dNK) cells. Therefore, these recruited CD16^-^ NK cells may be the actual precursors of dNK cells, which play important roles in spiral artery remodeling and placental vasculature formation. To date, it is unknown if HO-1 is involved in uterine NK (uNK) recruitment by SDF-1. Since significant reductions in uNK cells and in uNK-related angiogenic factors were indeed found in the HO-1-deficient (Het, HO-1^+/-^) placenta (Zenclussen et al., 2011; Zhao et al., 2011; Linzke et al., 2014), we speculate that HO-1 may have a regulatory role in mediating the crosstalk between EVTs and decidual stromal cells, including uNK cells (Du et al., 2012; Ren et al., 2012).

Expression of HO-1 in endothelial cells has been shown to also promote angiogenesis by downregulating anti-angiogenic mediators. Cudmore et al. (2007) demonstrated that adenoviral overexpression of HO-1 diminishes the production of anti-angiogenic sFlt1 receptor and sEng in response to the VEGF ligand in endothelial cells. In contrast, significantly higher levels of sFlt1 and sEng were found in HO-1-deficient compared to Wt mice. George et al. (2012) showed that HO-1 reduces hypoxia-induced sFlt-1 levels and oxidative stress in placental villi through CO and bilirubin. Since sFlt1 and sEng are key mediators in the pathogenesis of pre-eclampsia, the observation that HO-1 can suppress the release of sFlt1 and sEng was further investigated. Zhao et al. (2009) found that maternal plasma sFlt-1 levels, as well as diastolic blood pressures, were significantly elevated in pregnant HO-1 Het mice compared to Wt mice, suggesting that pregnant HO-1-deficient dams have characteristics similar to those in pre-eclampsia. Moreover, George et al. (2011a) also showed that an induction of HO-1 could alleviate sFlt-1-induced hypertension in pregnant rats.

The placenta has a complex and well-organized vascular network comprised of both maternal and fetal vessels. In early pregnancy, the uterine spiral arteries become remodeled into low resistance and high capacitance vessels, which is necessary to support the dynamically growing placenta and fetus. The association of HO-1 and uteroplacental vascular formation has been investigated by Zhao et al. (2011), Wong et al. (2012) using HO-1 Het mice. They collected placentas at E10.5, since this gestational age corresponds to the second trimester in human pregnancies, when the uteroplacental vasculature is becoming established. Histochemical staining revealed significant differences in the placental vasculature from HO-1 Het dams compared to pregnant Wt dams, such as thinner junction zones, a reduction in maternal sinusoidal number and size in the labyrinth, and a disorganization of fetal capillaries. These observations suggest that HO-1 deficiency is associated with vascular malformations in the labyrinth (Zhao et al., 2011). A vascular corrosion technique to cast placentas and CT imaging to reconstruct 3D images were used to visualize the vasculature of the placentas at ∼E16.5. They found that HO-1 Het placentas were smaller with a reduced labyrinth vessel volume and a thin maternal vascular region. Spiral arteries also appeared to be highly branched, but found to have smaller diameters (Zhao et al., 2011; Wong et al., 2012). They concluded that a partial deficiency of HO-1 leads to insufficient spiral artery remodeling and enlargement, which may by an underlying cause of pregnancy disorders, such as pre-eclampsia and intrauterine growth restriction (IUGR).

The formation of the uteroplacental vasculature network is a very complex event that involves not only endothelial cells, but also, trophoblast, uNK, decidual stromal, and infiltrating immune cells. These cells produce many cytokines, chemokines, growth factors, and angiogenic factors. Zhao et al. (2011) used PCR arrays to measure and then compare gene expression profiles from pregnant Wt and HO-1 Het uteri. Genes encoding for pro-angiogenic factors, such as growth factors, matrix metallopeptidase, and cytokines/chemokines, were significantly downregulated, while genes controlling anti-angiogenic factors were upregulated in HO-1 Het uteri. These data strongly suggest that the role of HO-1 in placental vasculature development is not due to a single event, but involves several cell types and the regulation of complex series of multiple steps.

Tumor angiogenesis initially begins with cancer cells releasing signaling molecules to the surrounding host tissues. These signals activate specific host genes that, in turn, stimulate the synthesis of proteins that stimulate new blood vessel growth. Unlike the tightly regulated and organized vasculature of the placenta, blood vessels of tumors are highly aberrant, dysfunctional, and uncontrolled. Despite the differences, the basic mechanism of tumor angiogenesis is very similar to those of uteroplacental vascular formation. HO-1 has also been suggested to be pro-angiogenic in many different tumor models, such as melanomas, pancreatic cancers, lung carcinomas, and gliomas (Was et al., 2010). By either inhibiting or inducing HO-1, researchers have found that HO-1 plays a critical role in mediating both VEGF-stimulated endothelial cell proliferation and SDF-1-induced EPC recruitment (Was et al., 2010).

Like that occurring in the placenta, the vascularization of tumors is also a complex process, relying not only on endothelial cells, but also on infiltrating immune cells, such as macrophages. *In vivo* studies have showed that an increased expression of HO-1 is associated with an augmented angiogenesis in human gliomas and melanomas. Mouse studies have shown that TAMs promote angiogenesis in tumors by both producing pro-angiogenic factors and physically assisting the sprouting of blood vessels. Further studies have shown that TAMs are comprised of phenotypically and functionally distinct subsets, including the pro-angiogenic (TIE2^+^) and the angiostatic/inflammatory (CD11c^+^) macrophages. The location and ratio of these two populations vary by the type and grade of the tumor (Welford et al., 2011). Since HO-1 is highly expressed in macrophages, it would be interesting to understand how HO-1 contributes to the differentiation and function of TAM subsets. In addition, macrophages are also observed in both deciduae and labyrinths. It may be of further interest to characterize whether macrophages in the placenta are pro-angiogenic or angiostatic, and to investigate their function in uteroplacental vascular formation.

## HO-1 IMMUNOMODULATION AND FACILITATION OF IMMUNOTOLERANCE AND IMMUNE PRIVILEGE

During early pregnancy, fetal-derived trophoblast cells, especially in EVTs, penetrate deep into the uterine wall to form the decidua. At the same time, many maternal leukocytes, including monocytes, macrophages, dendritic cells (DCs), NK cells, neutrophils, and regulatory T cells (Tregs), also infiltrate into the decidua in order to support early placental development. Interestingly, direct contact of the semi-allogeneic EVTs with maternal leukocytes and decidual stromal cells does not induce rejection, but instead results in immune privilege. Initial studies suggest that prevention of fetal rejection is associated with an increased ratio of T helper type 2 (Th2) compared to Th1 cytokines produced by maternal leukocytes. However, a wealth of follow-up studies indicates that tolerance mechanisms initiated here are much more complicated than initially thought. It appears to involve the adaption of both innate and adaptive immune responses, locally, and systemically, aided by endocrine pathways (Arck and Hecher, 2013; Erlebacher, 2013).

HO-1 has been long accepted as an anti-inflammatory and immunosuppressive molecule. Its induction by pharmacological activators, through gene transfer, and delivery of heme metabolites (CO and bilirubin) produce immunosuppressive effects in a variety of conditions or disorders, such as autoimmune disease and organ transplantation (Becker et al., 2007; Tzima et al., 2009). HO-1 is expressed in circulating monocytes and tissue macrophages. In HO-1 knockout mice, residential macrophages such as spleen sinusoidal lining cells and liver Kupffer cells, are absent (Kovtunovych et al., 2010); while adoptively transferring Wt macrophages to HO-1-deficient mice has been shown to reverse disease (Kovtunovych et al., 2014), suggesting an important role of HO-1 in macrophage survival and function. Lee and Chau (2002) reported that HO-1 in macrophages mediates the anti-inflammatory function of IL-10, a classic Th2 cytokine. Inhibition of HO-1 protein or HO activity significantly reverses the inhibitory effect of IL-10 on the production of tumor necrosis factor-alpha (TNF-α) induced by lipopolysaccharide (LPS; Lee and Chau, 2002).

The adverse effects of HO-1 deficiency related to immune regulation during pregnancy are mostly observed in the context of human and murine spontaneous abortions. It has been reported that women with early miscarriages are prone to have longer (GT)n repeats in the HO-1 promoter region, and thus a lower expression of HO-1 (Denschlag et al., 2004). Zenclussen et al. (2002) has used two different models to elucidate the importance of HO-1 in spontaneous abortions. HO-1 levels in placentas and deciduae were reduced in mice with sonic stress or IL-12-induced abortions. When HO-1 was delivered to spontaneous abortion mice via adenoviral transfer, rates of abortion declined with an increase of the IL-4 to interferon-gamma (IFN-γ) ratio in spleen lymphocytes and a decrease in apoptosis at the fetomaternal interface (Zenclussen et al., 2006b). Similarly, induction of HO activity can increase the expression of Bag-1, an anti-apoptotic factor, at the fetomaternal interface as well as activate Tregs, to collectively result in a lower spontaneous abortion rate (Zenclussen et al., 2005). Inhibition of HO activity by treatment with zinc protoporphyrin (ZnPP) not only increased abortion rates in normal as well as abortion-prone pregnancies; but also, was found to abolish the protective effects of the transfer of Tregs (Schumacher et al., 2012). In addition, transfer of Tregs from healthy pregnant mice into abortion-prone mice reduced fetal rejection rates and resulted in the upregulation of HO-1 and TGF-β at the fetomaternal interface (Zenclussen et al., 2006a). Even with so much evidence showing the importance of HO-1 in the maintenance of a normal pregnancy, the exact mechanisms responsible for immunoregulation in the fetomaternal interface have not been fully elucidated.

**FIGURE 2:**
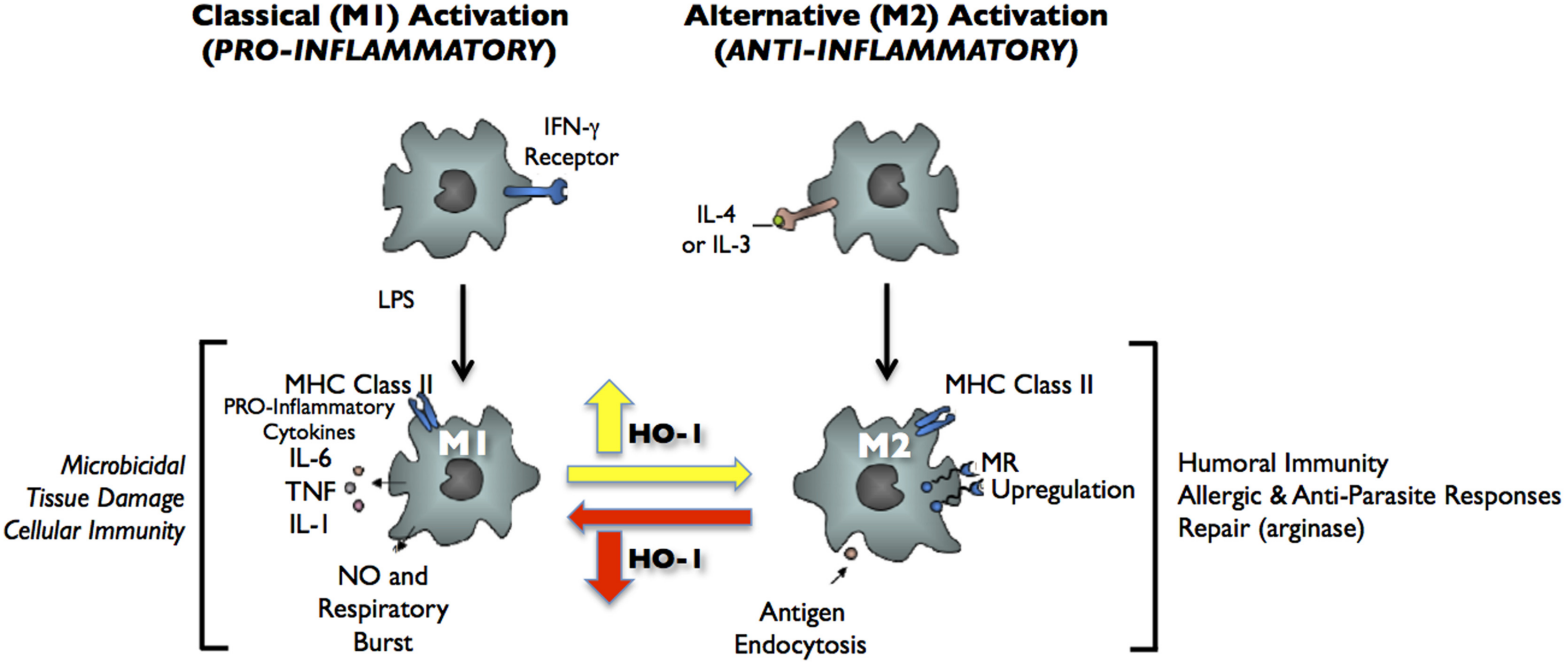
**Classical activation (M1) and alternative activation (M2) of macrophages.** Classical activation is mediated by the priming stimulus IFN-γ, followed by a microbial trigger (lipopolysaccharide, LPS). Alternative activation is mediated by IL- 4 or IL-13. The uptake of apoptotic cells or lysosomal storage of host molecules generates anti-inflammatory responses. Cytokines (IL-10, TGF-β, IFN-α/β) are potent modulators of activation.

Decidual macrophages are present throughout gestation and reside near the cytotrophoblast shell in close contact with EVTs. Decidual macrophages phagocytose apoptotic EVTs and secrete IL-10 and indoleamine 2,3-dioxygenase (IDO), adding to the tolerogenic Th2 environment. Gene expression profiling showed that decidual macrophages are immunosuppressive and anti-inflammatory with high expression of CCL18, CD206, insulin-like growth factor (IGF-1), IDO, and other genes associated with M2-polarized macrophages (Gustafsson et al., 2008). Recent evidence suggests that HO-1 is critically involved in macrophage polarization to the M2 phenotype (Weis et al., 2009; Choi et al., 2010; Sierra-Filardi et al., 2010; **Figure 2**). HO-1 expression levels are significantly higher in M2 macrophages and induction of HO-1 can significantly enhance IL-10 production, while it has no effect on M1 cytokine production (Sierra-Filardi et al., 2010). More interestingly, HO-1 may mediate the M1 to M2 phenotypic switch when mice are treated with heme (Choi et al., 2010). Further investigations are needed to best understand the function of HO-1 in decidual macrophages and how HO-1 contributes to placental development.

Dendritic cells have also been found in mouse and human deciduae, and undergo a restricted T cell response to a fetal specific-antigen. Most decidual DCs (dDCs) remain in an immature and tolerogenic state (tDCs). These cells exhibit an altered capacity for antigen presentation, with reduced expression of co-stimulatory molecules and IL-12, but an enhanced production of IL-10. tDCs can promote immunotolerance by inducing effector T cell apoptosis and expansion of CD4^+^ Tregs. In addition, even in syngeneic mouse pregnancies, depletion of uterine DCs causes failure of decidualization and resorption of embryos, suggesting that a potentially more complex role for DCs in the development of the fetomaternal interface other than just antigen presentation and secretion of immunosuppressive cytokines. Moreover, HO-1 contributes to the maintenance of immature and anti-inflammatory state tDCs. Chauveau et al. (2005) have shown that HO-1 is only expressed in immature tDCs, but not in mature DCs. HO-1 induction in mature DCs lead to a loss of DC immunogenicity and pro-inflammatory function. Moreau et al. (2009) reported that HO-1 was required for tDCs to delay cardiac allograft rejection in the rat. Park et al. (2010) showed that expression of HO-1 by DCs is necessary for CD4^+^ Tregs to exert their immunoregulatory activity. Although DCs are well studied in pregnant uteri, the roles of HO-1 in the differentiation and function of dDCs are still not yet fully understood.

Tregs (CD4^+^CD25^+^foxp3^+^) contribute to the implantation and maintenance of early pregnancy via their immunosuppressive and tolerance properties. Many studies have reported that the frequency of Tregs in mice and human deciduae increases during healthy pregnancies, while they decrease in abortion-prone pregnancies (Erlebacher, 2013). Rowe et al. (2012) found that in murine pregnancies the number of Tregs double, while Tregs specific to fetal antigens expand more than 100-fold. Although HO-1 is present in Tregs, the actual function of HO-1 in Tregs has been controversial. One study reported that the regulatory function of Tregs is lost when HO activity was abolished by a pharmacological inhibitor (Andersen et al., 2009), while another study demonstrated that an amplification of the Treg population following the same treatment (Biburger et al., 2010). Studies from HO-1^-/-^ mice have provided a definitive answer: HO-1 expression in Tregs is not required for Treg immunosuppression (Zelenay et al., 2007); instead, Treg function is likely to be affected indirectly by HO-1 expression in antigen-presenting cells (APCs), such as DCs or macrophages (George et al., 2008). Indeed, Schumacher et al. (2012) found that HO-1 indirectly contributes to the expansion of the peripheral Treg population by maintaining maternal DCs in an immature state.

Similar to trophoblasts in pregnancy, malignant cells can express antigens that mediate recognition by host CD8^+^ T cells. These antigens are mostly the result of a point mutation in normal genes. Cancer cells may have hundreds or even thousands of mutations in their coding exons, contributing a large repertoire of antigens that can serve as potential immune targets. Despite an abundance of antigens, most cancers can still progress and evade attack by the immune system. This phenomenon of “tumor escape” may be due to immunosuppression and tolerance in the tumor microenvironment.

Most solid tumors consist of many types of infiltrating leukocytes, including macrophages, DCs, neutrophils, NK cells, and T and B cells. Interestingly, very similar to the immune microenvironment in the fetomaternal interface, these infiltrating cells are mostly polarized toward immunosuppressive and tumor-promoting phenotypes. For example, TAMs are the most abundant population and display an M2-like phenotype. They promote tumor angiogenesis, cell invasion and metastasis, and facilitate cytoprotection from chemotherapy-induced apoptosis. They exhibit an ineffective ability to present antigens, but can attract Tregs to inhibit T cell activation. Similarly, DCs in tumors are also immature and functionally incompetent with a tolerance phenotype. Cancer cells secrete substances, such as VEGF, TGF-β, hepatocyte growth factor, and osteopontin, that can suppress the maturation of DCs (Holtan et al., 2009). These immature, tDCs express high levels of the pro-angiogenic cytokine VEGF, the pro-inflammatory cytokines IL-6 and IL-8, the immunosuppressive mediators IL-10, cyclo-oxygenase 2 (COX2), TGF-β, and IDO. Besides macrophages and DCs, immature neutrophils, also called myeloid-derived suppressor cells (MDSCs), display suppressive functions of T and NK cells in the tumor site (Gabrilovich and Nagaraj, 2009).

Arnold et al. (2014) demonstrated tumor immune suppression by macrophages expressing high levels of HO-1. However, the mechanisms of HO-1 on immunotolerance employed by macrophages, DCs, and MDSCs have not been fully elucidated. One possibility is through CO, a metabolite of heme degradation. It has been shown that CO promotes the development of tDCs (Remy et al., 2009) and inhibits T cell proliferation (Song et al., 2004). In addition, Zenclussen et al. (2011) and Linzke et al. (2014) have reported that CO inhalation successfully restores the Wt phenotype in HO-1 Het pregnancies. Another putative mechanism of HO-1 may be mediated through other heme binding enzymes, such as IDO (Curti et al., 2009) and NADPH oxidase (Ryter et al., 2006). IDO, a heme binding and tryptophan catabolic enzyme, has been suggested to be the key player involved in the inhibition of cell proliferation and induction of immunotolerance during infection, pregnancy, transplantation, autoimmunity, and hematologic malignancies (Curti et al., 2009). Jung et al. (2010) found that murine DC maturation depends on IDO expression via a HO-1-dependent pathway. They found that in IDO deficiency, there is a loss of maturation of DCs *in vitro* and *in vivo*. In addition, inhibition of HO by ZnPP abolished IDO expression and DC maturation, while the administration resulted in opposite effects. Therefore, studying the immunosuppressive function of leukocytes in the fetomaternal interface as well as in the tumor site may help reveal the role of HO-1 in immune regulation (Blancou and Anegon, 2010; Gabrilovich et al., 2012).

## CONCLUSION

HO-1 is a ubiquitous stress-response gene that is expressed and induced in a large variety of cell types. In the fetomaternal interface, HO-1 is present in several cells, such as trophoblasts (especially EVTs), endothelial cells, and infiltrating leukocytes. Accordingly, HO-1 facilitates multiple functions during the establishment of the fetomaternal interface, which include cytoprotection, trophoblast invasion, pro-angiogenesis, and immune regulation. A deficiency of HO-1 results in pregnancy failure. Similarly, HO-1 is also expressed in malignant cells and infiltrating leukocytes in tumor sites, and shares comparable biological functions to promote tumor progression as those observed during placental development. Teasing out the function of HO-1 in each cell type may prove challenging and intriguing; but if successful, would greatly enhance our understanding of HO-1 biology as well as of the complex physiological and pathological processes of pregnancy and tumor formation. The implications of the similarities of the two systems will help in the design of integrated approaches to study pregnancy and cancer.

## Conflict of Interest Statement

The authors declare that the research was conducted in the absence of any commercial or financial relationships that could be construed as a potential conflict of interest.
